# Fitness to fly for children and adolescents after Fontan palliation

**DOI:** 10.3389/fcvm.2023.1170275

**Published:** 2023-06-23

**Authors:** N. Müller, U. Herberg, J. Breuer, T. Kratz, J. A. Härtel

**Affiliations:** ^1^ Department for Pediatric Cardiology, Children’s Heart Center UK Bonn, University Hospital Bonn, Bonn, Germany; ^2^ Department for Pediatric Cardiology, University Hospital Aachen, Aachen, Germany

**Keywords:** children and adolescents, Fontan circulation, fitness to fly, hypoxic challenge test, normobaric hypoxia

## Abstract

**Introduction:**

At cruising altitude, the cabin pressure of passenger aircraft needs to be adjusted and, therefore, the oxygen content is equivalent to ambient air at 2,500 masl, causing mild desaturation and a rising pulmonary vascular resistance (PVR) in healthy subjects. For Fontan patients with passive pulmonary perfusion, a rising PVR can cause serious medical problems. The purpose of this fitness to fly investigation (FTF) is to assess the risk of air travel for children and adolescents after Fontan palliation.

**Methods:**

We investigated 21 Fontan patients [3–14y] in a normobaric hypoxic chamber at a simulated altitude of 2,500 m for 3 h. Oxygen saturation, heart rate, and regional tissue saturation in the forehead (NIRS) were measured continuously. Before entering the chamber, after 90 and 180 min in the hypoxic environment, blood gas analysis and echocardiography were performed.

**Results:**

Heart rate and blood pressure did not show significant intraindividual changes. Capillary oxygen saturation (SaO_2_) decreased significantly after 90 min by a mean of 5.6 ± 2.87% without further decline. Lactate, pH, base excess, and tissue saturation in the frontal brain did not reach any critical values. In the case of open fenestration between the tunnel and the atrium delta, P did not increase, indicating stable pulmonary artery pressure.

**Conclusion:**

All 21 children finished the investigation successfully without any adverse events, so flying short distance seems to be safe for most Fontan patients with good current health status. As the baseline oxygen saturation does not allow prediction of the maximum extent of desaturation and adaption to a hypoxic environment takes up to 180 min, the so-called hypoxic challenge test is not sufficient for these patients. Performing an FTF examination over a period of 180 min allows for risk assessment and provides safety to the patients and their families, as well as the airline companies.

## 1. Introduction

Caused by the unique physiology of passive pulmonary perfusion due to a lacking subpulmonary ventricle, treating patients with Fontan palliation has several special issues (1). This must be taken into account for the planning of everyday life and leisure activities. Recently published data estimated a rising number of people living with Fontan circulation from approximately 48,000 in 2020 to 60,000 in 2030 including 11 countries across Europe, USA, Australia, and New Zeeland with an increasing number of adult patients (55%–64%) (2).

Due to the increasingly better general condition of children after Fontan surgery, the treating pediatric cardiologists have to take a stand on questions such as permission to travel by air. In terms of the psychological aspect of living with Fontan circulation and its association with elevated symptoms of depression as a negative predictor for quality of life, restrictions should be reduced to a minimum (3).

The barometric pressure within the cabin of a passenger aircraft is equivalent to a maximum of 2,438 m above sea level (masl) (8,000 ft). This is regulated by the European and North American authorities for normal operating conditions (4). The barometric pressure at a cruising altitude leads to a lower partial pressure of inspired oxygen equivalent to 15.2% ambient oxygen, resulting in lower transcutaneous oxygen saturation (SpO_2_) in healthy children and adults down to 94.4% (5, 6) and 88%–94% in term and preterm infants (7). Partial oxygen pressure (pO_2_) is the major regulator of pulmonary vascular tone and a fall in alveolar pO_2_ is the main stimulus for hypoxic pulmonary vasoconstriction (HPV). A rising pulmonary artery pressure (PAP) is an early and inevitable consequence of ascent to high altitude in humans with biventricular hearts (8). The level of altitude has an inverse relation to arterial oxygen saturation (SaO_2_) and a direct relationship to the PAP (9).

In Fontan circulation, where the blood flow is maintained mainly by respiration and the central venous pressure (CVP), a linear relationship between CVP and pulmonary vascular resistance (PVR) exists (10). A mild increase in PVR might already impair cardiac output (CO).

To assess the risk for patients with relevant underlying diseases before they board an aircraft, hypoxic challenge tests (HCT) have been established. By breathing oxygen-depleted air (15.2%) for a period of 5–20 min (7, 11–13), usually sitting in an upright position in a body plethysmograph (11) or breathing via a face mask (5), an approximate similar inspired pO_2_ to breathing air at cruising altitude can be simulated under safe conditions.

The British Thoracic Society (BTS) already recommends HCT for specific constellations to determine whether supplemental in-flight oxygen is necessary or medical clearance can be given (4).

For Fontan patients, especially for those with a lower baseline oxygen saturation due to fenestration of the Fontan tunnel or pronounced collaterals, an evaluation of the pre-flight condition appears to be beneficial. Spoorenberg et al. investigated children and adolescents with congenital heart disease performing HCT, integrating changing body positions and mild physical activity. In this study, only two patients with Fontan circulation were included (14). Morimoto et al. exclusively investigated 11 Fontan patients and eight volunteers with a mean age of 22 years under real flight conditions on two-hour flights measuring percutaneous oxygen saturation (SpO_2_). He described lower baseline SpO_2_ in Fontan patients and a significant reduction of SpO_2_ after ascent and, more importantly, during cruise after 1 h compared to healthy controls (15). This shows that not only for children and adolescents with a Fontan circulation but also ex-preterm babies with or without bronchopulmonary dysplasia, adults with cyanotic congenital heart disease, passengers with cardiovascular disease, or patients with pulmonary hypertension, fitness to fly investigation is an issue of great importance (5, 7, 12, 16–18) and may be superior to HCT testing.

The aim of our study was to investigate Fontan patients for fitness to fly under safe conditions at a simulated altitude in a normobaric altitude chamber with SpO_2_, capillary blood gas analysis (BGA), regional brain oximetry on the forehead (rSO_2_), and echocardiography in order to provide a better assessment of altitude-related changes over a longer period of time.

## 2. Materials and methods

### 2.1. Participants

We investigated 21 children and adolescents after the Fontan procedure with different types of underlying anatomical heart diseases (Table 1). Participants were selected from the hospital’s database and asked whether they were interested in participating in the study. The patients and their legal guardians received information material with the specific study design and gave their written informed consent before participation.

**Table 1:** Patients characteristics, * = age as mean and [min; max].

		Fontan (*n = 21)*
Age	Years	8.7 [3.2; 14.7]*
Gender	f/m	7/14
Height	cm	131.12 ± 20.29
Body mass	kg	29.79 ± 11.83
Body-mass-index	kg/m^2^	16.68 ± 2.35
Body surface area (Mosteller)	m^2^	1.03 ± 0.28
Age at Fontan completion	Month	36.8 ± 9.7
Years since Fontan surgery	Years	5.7 ± 3.2
Patients with implanted pacemakers		2
Patients with fenestration		7
Underlying cardiac defect	I. Functional left ventricle	
	Tricuspid atresia	3
	Double Inlet Left Ventricle (DILV)	2
	Pulmonary atresia	1
	II. Functional right ventricle	
	Hypoplastic left heart syndrome	7
	Double Outlet Right Ventricle (DORV)	4
	Pulmonary atresia with TGA	2
	III. Indeterminate	
	Atrioventricular canal defect	1
	Shone’s complex	1
Medication	I. PDE-5-inhibitors (Sildenafil)	3
	II. Cardioselective β-Blocker	6
	III. ACE-inhibitors	11

The sample size of 21 participants was chosen as the number of at least 20 subjects ensures that events (problems and risks) that occur in at least 10% of cases can be observed at least once in the study population with a probability of 85%.

Patients >3 years with a minimum interval to the Fontan operation of 6 months and no contraindications for a stay at altitude (e.g., severe pulmonary hypertension) were included. Exclusion criteria were defined as baseline saturation below 85%, symptoms of failing Fontan-like plastic bronchitis or protein-losing enteropathy within the last 6 months, pulmonary hemorrhage within the last 6 months, acute infection at the time of the examination, or a withdrawn declaration of consent (by the patient or their legal guardians). As the topic of fitness to fly is equally interesting for all families, patients with an open fenestration in the Fontan tunnel (*n* = 7) or patients under therapy with PDE-5 inhibitors (*n* = 3) were deliberately not excluded from the study.

Approval was obtained from the local ethics committee of the University Hospital Bonn (application number 215/19) and the study corresponded with the Declaration of Helsinki.

The study was performed at the University Children’s Hospital Bonn, and data were collected from November 2019 to November 2021.

### 2.2. Study procedure

#### 2.2.1. Study design

A three-hour period was chosen for investigation in terms of getting stable conditions after adaptation to hypoxia. Additionally, most European holiday regions can be reached within this flight distance. *All investigations started at nine am in the morning*.

The time points for the evaluation were defined as Normoxia (outside the chamber): T0 = baseline; Hypoxia (inside the chamber): T1 = 30 min., T2 = 60 min., T3 = 90 min., T4 = 120 min., T5 = 150 min., and T6 = 180 min.; Normoxia (outside the chamber): T7 = 15 min. after termination of hypoxia.

The patients were encouraged to drink at least one liter of liquid during the experiment. If necessary, a mobile toilet could be used in the chamber, so this did not interrupt the exposure to hypoxia. During the examination, families were asked to behave as they would if traveling by plane, playing games, reading, or doing homework. The activity level of the children was tried to be limited by the parents to a low level. All participants were asked to avoid sleeping during the experiment as this is already known to cause a change in breathing patterns in healthy subjects and thus influences the saturation.

##### 2.2.1.1. Equipment

Continuous SpO_2_ and heart rate as well as intermittent blood pressure were displayed by CARESCAPE V100(GE Healthcare, Chicago/USA).

Continuous brain oximetry was measured by Near InfraRed Spectroscopy (NIRS, SenSmart X-100 Universal Oximetry System, Terumo Cardiovascular, Ann Arbor/USA) with EQUANOX rSO_2_ Optode on the forehead starting right after entering the chamber.

The blood gases were analyzed with Siemens Healthineers RAPIDLab™ 1265 Blood Gas Analyzer and nonheparinized capillary tubes.

The flight simulation was performed in a fully climatized normobaric altitude chamber (Höhenbalance, Going/Austria) with stable temperature conditions between 21 and 22.5°C. The chamber had 12 m^2^, containing approximately 37.5 m^3^ of air. Hourly, 60 m^3^ of air was refreshed. Ambient oxygen was reduced by membrane processes to a level of 15.2% (±0.2%) and replaced by nitrogen (84.8%) at constant air pressure (≈1,013 hPa), simulating an altitude of 2,500 masl (corresponding to Boyle’s law, estimating a barometric pressure of 760 hPa at 2,500 m). When CO_2_ increased over 0.2%, fresh air was automatically ducted into the altitude chamber. The hypoxic chamber was started approximately 2 h in advance to get a stable condition. The oxygen content in the chamber was measured using an electrochemical oxygen partial pressure sensor (GOX100, GMH Messtechnik GmbH, Greisinger, Regenstauf/Germany). Since the upper part of the chamber consists of glass elements all around, the examiners can stay outside the chamber, document the measured values, and observe the patient clinically. The chamber can be entered through a regular door without relevant changes in oxygen content inside so that medical staff can switch between the chamber and the outside area for the necessary examinations.

Echocardiography at T0, T3, and T6 was performed and analyzed with a Philips IE33 ultrasound system. Velocity time integral (VTI) was calculated by multiplication with heart rate as a surrogate of CO (CO = VTI*HR).

##### 2.2.1.2. Baseline data (T0, normoxia)

The baseline data were collected in a separate room at a sufficient distance from the altitude chamber in ambient air with 21% oxygen. Heart rate, blood pressure, and SpO_2_ were measured in an upright sitting position followed by the initial echocardiography in a supine or left lateral position. The sample for the first capillary blood gas analysis was taken in a sitting or lying position from the fingertip using safety lancets with a penetration depth of 0.85 or 1 mm. The optode for rSO_2_ (NIRS) was placed on the forehead.

##### 2.2.1.3. Flight investigation (T1–T6, hypoxia)

After the patient and one accompanying person (mostly parents) entered the hypoxic chamber, the monitoring was set up and the three-hour investigation time was started.

Heart rate, blood pressure, transcutaneous oxygen saturation (SpO_2_), and regional oxygen saturation (rSO_2_) were constantly measured and documented every 30 min in an upright sitting position. After 90 (T3) and 180 min (T6) in hypoxia, an echocardiography was performed inside the chamber, again lying on the examination couch in supine or left lateral positions. The measurements started 3 min after changing to a supine position. Blood gases were drawn while lying or sitting on the examination couch after the echocardiography was performed. The investigation ended after 180 min (T6) in the hypoxic environment by opening the door and stopping the supply of oxygen-depleted air.

##### 2.2.1.4. Post-hypoxia period (T7)

Another probe for BGA was taken 15 min after the termination of hypoxia and the removal of all electrodes and measurement equipment.

#### 2.2.2. Statistical analysis

GraphPad Prism (V. 9.4.0, GraphPad Software, San Diego, CA/USA) was used for statistical analysis.

Continuous outcomes were assessed for normality using the D’Agostino and Pearson test.

Variables fitting a normal distribution were tested on differences using one-way repeated measures ANOVA and Dunnett’s multiple comparison tests for post-hoc analysis. Otherwise, the Friedman test was applied.

Participants with missing data were excluded from the analysis but were shown in descriptive data. When data were missing, the number of included values was reported in the tables. For blood gas analysis, 5% of participants’ data were missing at T0, T3, T6, and 62% at T7.

Simple linear regression was performed for correlation analysis between parameters, and Pearson’s correlation coefficient was applied to measure correlation strength.

Quantitative data were presented in mean ± standard deviation (SD) unless otherwise described.

*p*-values ≤ 0.05 (two-sided) were defined as statistically significant and corresponding values in tables and figures are presented as “*”.

## 3. Results

### 3.1. Clinical parameters

All participants were able to complete the examination. An early termination due to clinical symptoms was not necessary. A six-year-old boy was pre-syncopal twice after blood gas analysis (before entering the chamber and at T3) but also finished the examination since this was a known problem for him. In addition, a 12-year-old girl complained of dizziness over a period of 10 min after 1.5 h of hypoxia. Vital signs were stable at that time, and the dizziness resolved spontaneously after distraction and oral fluid intake. The remaining 19 patients did not feel any changes and did not show any clinical signs or make complaints of symptoms. At the end of the hypoxia period, the children under 10 years of age, in particular, were exhausted and tired, which was manifested by resentment, crying, and clinical symptoms such as yawning and lack of movement. Due to the young age of the participants and the large age range, an objectification of this condition by means of a questionnaire or similar was not reasonably possible but could be evaluated in conversation with the parents.

**Figure 1:**
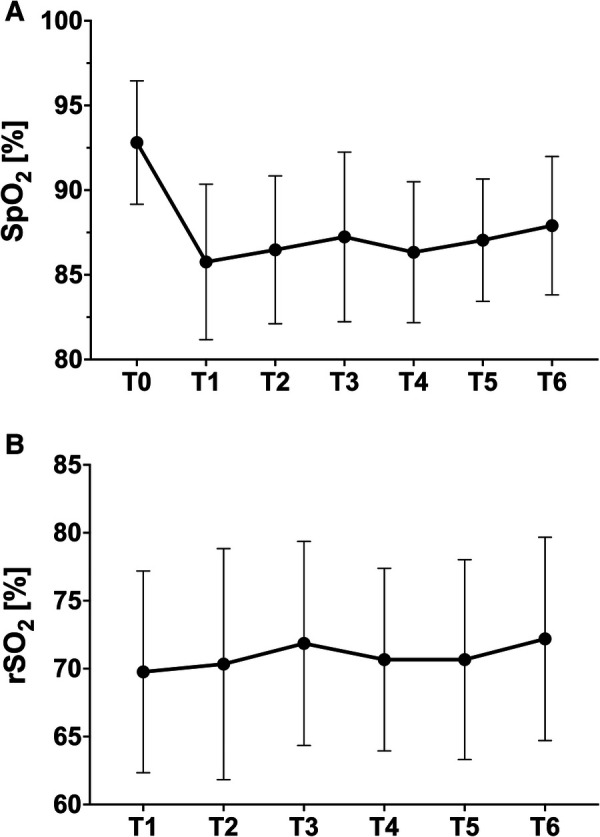
(**A**) Peripheral oxygenation (finger); (**B**) tissue oxygenation (NIRS forehead) at different time points (mean ± standard deviation).

All measured clinical parameters are listed in Table 2A. Heart rate and blood pressure did not show significant intraindividual changes. SpO_2_ dropped significantly after entering the hypoxic environment with a nadir 30 min after the start of altitude exposure (mean delta T0-T1: 6 ± 3.8%). All patients showed an increase in peripheral oxygen saturation during the course after the initial drop without changes in the conditions (mean delta T1–T6: 2 ± 2.4%) (Figure 1A).

**Table 2:** (**A**) clinical parameters; (**B**) blood gas analysis; *p*-value 1(T0–T3), *p*-value 2 (T0–T6), *p*-value 3 (T0–T7).

(A)		T0	T1	T2	T3	T4	T5	T6
Heart rate	[bpm]	86 ± 15	88 ± 13	95 ± 14	90 ± 14	97 ± 13	97 ± 13	94 ± 18
SpO_2_	[%]	93 ± 4	86 ± 5	86 ± 4	87 ± 5	86 ± 4	87 ± 4	88 ± 4
rSO_2_	[%]	/	70 ± 7	70 ± 8	72 ± 8	71 ± 7	71 ± 7	72 ± 7
RRsys	[mmHg]	108 ± 15	106 ± 16	109 ± 14	111 ± 13	110 ± 11	107 ± 11	110 ± 14
RRdia	[mmHg]	63 ± 9	62 ± 11	62 ± 9	68 ± 11	63 ± 9	62 ± 12	65 ± 12
(B)		T0 (*n* = 20)	T3 (*n* = 20)	T6 (*n* = 20)	T7 (*n* = 8)	*p*-value 1	*p*-value 2	*p*-value 3
pH		7.44 ± 0.02	7.45 ± 0.02	7.45 ± 0.02	7.44 ± 0.03	ns	0.018*	ns
pCO_2_	[mmHg]	32.25 ± 6.8	29.13 ± 2.2	28.98 ± 2.51	29.2 ± 2.87	0.001*	0.002*	ns
pO_2_	[mmHg]	59.87 ± 7.99	49.98 ± 4.37	49.6 ± 4.59	59.59 ± 9.19	<0.001*	<0.001*	ns
HCO_3_ act	[mmol/l]	20.41 ± 1.13	19.87 ± 1.27	19.9 ± 1.31	19.29 ± 1.37	0.008*	0.016*	0.023*
HCO_3_ std	[mmol/l]	22.09 ± 0.71	21.92 ± 1.1	21.92 ± 0.89	21.38 ± 1.06	ns	ns	ns
Base Excess	[mmol/l]	−2.68 ± 0.83	−2.93 ± 1.1	−2.72 ± 1.07	−3.6 ± 1.25	ns	ns	ns
Lactate	[mmol/l]	1.69 ± 0.37	2.18 ± 0.64	2.14 ± 0.55	2.3 ± 0.83	0.011*	0.029*	ns
Hemoglobin	[g/dl]	13.55 ± 1.13	13.68 ± 1.12	13.61 ± 1.25	13.43 ± 1.6	0.049*	ns	ns
SaO_2_	[%]	90 ± 4	85 ± 4	85 ± 5	91 ± 4	<0.001*	<0.001*	ns

* indicates statistical significance.

The youngest patient with 7 months after Fontan surgery (without fenestration but with a large IVC to atrium shunt) and the oldest patient, 11 years post-Fontan completion (with fenestration) showed the highest drop in oxygen saturation with a loss of 15% from T0 to T1. The young one started with SpO_2_ of 88% before entering the chamber then dropping to 73% at T2 and recovering after T3 with peripheral saturation values around 80%–82%. In the blood gas analysis, a SaO_2_ of 81.2% was measured at T3. The patient’s clinical condition was unimpaired at all times. The oldest patient started with 96% SpO_2_, desaturated to 81% at T1, again recovering after T3 with 86%–87%. The SaO_2_ at T3 showed significantly higher values of 88.9%.

In contrast, in all patients, a constant tissue saturation of the brain could be derived via the NIRS optodes on the forehead also, with a trend of improvement after 60 min. in hypoxia (Figure 1B).

There was no correlation between SaO_2_ and HR or rSO_2_.

### 3.2. Blood gas analysis

Repeated capillary blood gases from the finger were tolerated even by the smaller patients. At T7, most children were desperate to finish the investigation and did not allow a fourth capillary blood sampling. In order not to traumatize them and associate flying with negative experiences, sampling was not encouraged (Table 2B).

**Figure 2:**
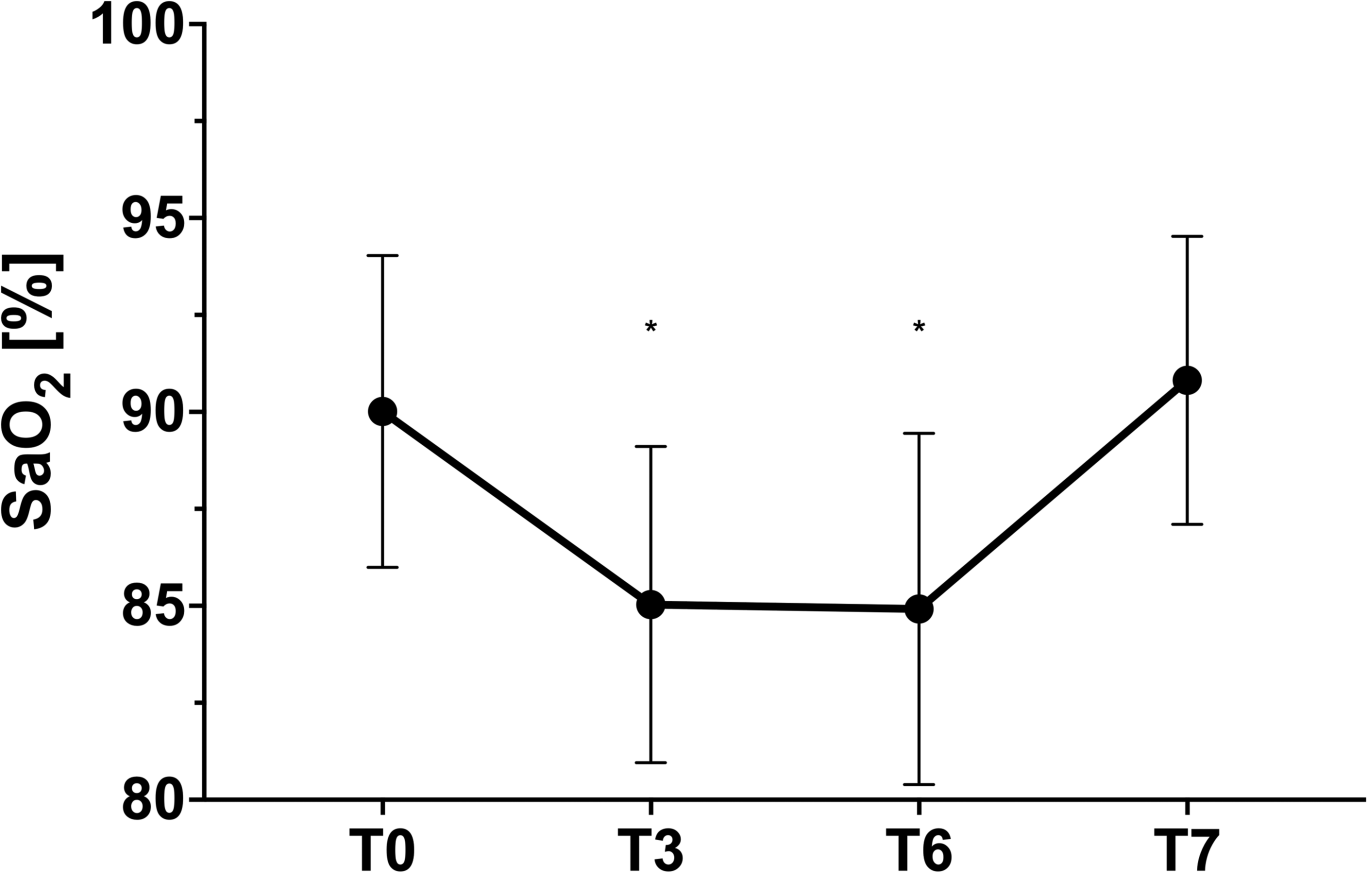
Capillary SaO_2_, * = *p* < 0.05 in relation to T0.

In the blood gas analysis, capillary oxygen saturation after 90 min showed a significant decrease by a mean of 5.6 ± 2.87% but did not drop further during the course in the majority of the children (Figure 2). pO_2_, on the other hand, showed a further decline from T3 to T6 with complete recovery at T7 after exiting the hypoxic environment (Table 2B).

All over, the mean difference between the transcutaneous SpO_2_ and the capillary SaO_2_ was −2.6 ± 2.9%. A good correlation with SpO_2 _≤ 0.0001, *R*^2^ = 0.6715 could be shown.

CO_2_ decreased while base excess, bicarbonate, and pH remained stable without significant changes. Lactate increased significantly from T0 to T3, without an additional rise at T6 and a further but non-significant increase up to T7 (here with a greater range) (Table 2B).

### 3.3. Echocardiography

Table 3 highlights the parameters being influenced by hypoxia. IVC Vmax, IVC MPG, Vmax at aortic arch, and E/É medial and lateral were also recorded but not presented due to lack of relevance to the research question or incomplete data sets caused by the very different anatomical conditions.

**Table 3:** Echocardiographic parameters; *p*-value 1 (T0–T3), *p*-value 2 (T0–T6), * = *p* < 0.05.

		T0	T3	T6	*p*-value 1	*p*-value 2
ΔP_fenestration_	[mmHg]	5.4 ± 1.1 (7)	5.7 ± 1.2 (6)	5.7 ± 1.7 (7)	ns	ns
VTI_A0_		23 ± 4.2	22.4 ± 4.3	21.6 ± 3.9	ns	ns
CO	[VTI × HR]	1,920 ± 392	2,016 ± 463	2,027 ± 557	ns	ns
AV E Vmax	[cm/s]	87.20 ± 17	78.10 ± 25.90	74.40 ± 16.10	ns	0.006*
AV A Vmax	[cm/s]	57.40 ± 14.70	56.60 ± 12.5	55.40 ± 13.40	ns	ns
AV E/A		1.6 ± 0.3	1.5 ± 0.3	1.4 ± 0.2	ns	0.043*

Regarding the AV-valve inflow, significant changes could be monitored with a reduction in AV E Vmax and a lacking decrease of the a-wave leading to a reduction of AV E/A indicating diastolic function.

The VTI as a marker for cardiac function showed a slight but not significant decrease. CO did not change significantly from T0 to T6 (Table 3).

**Figure 3:**
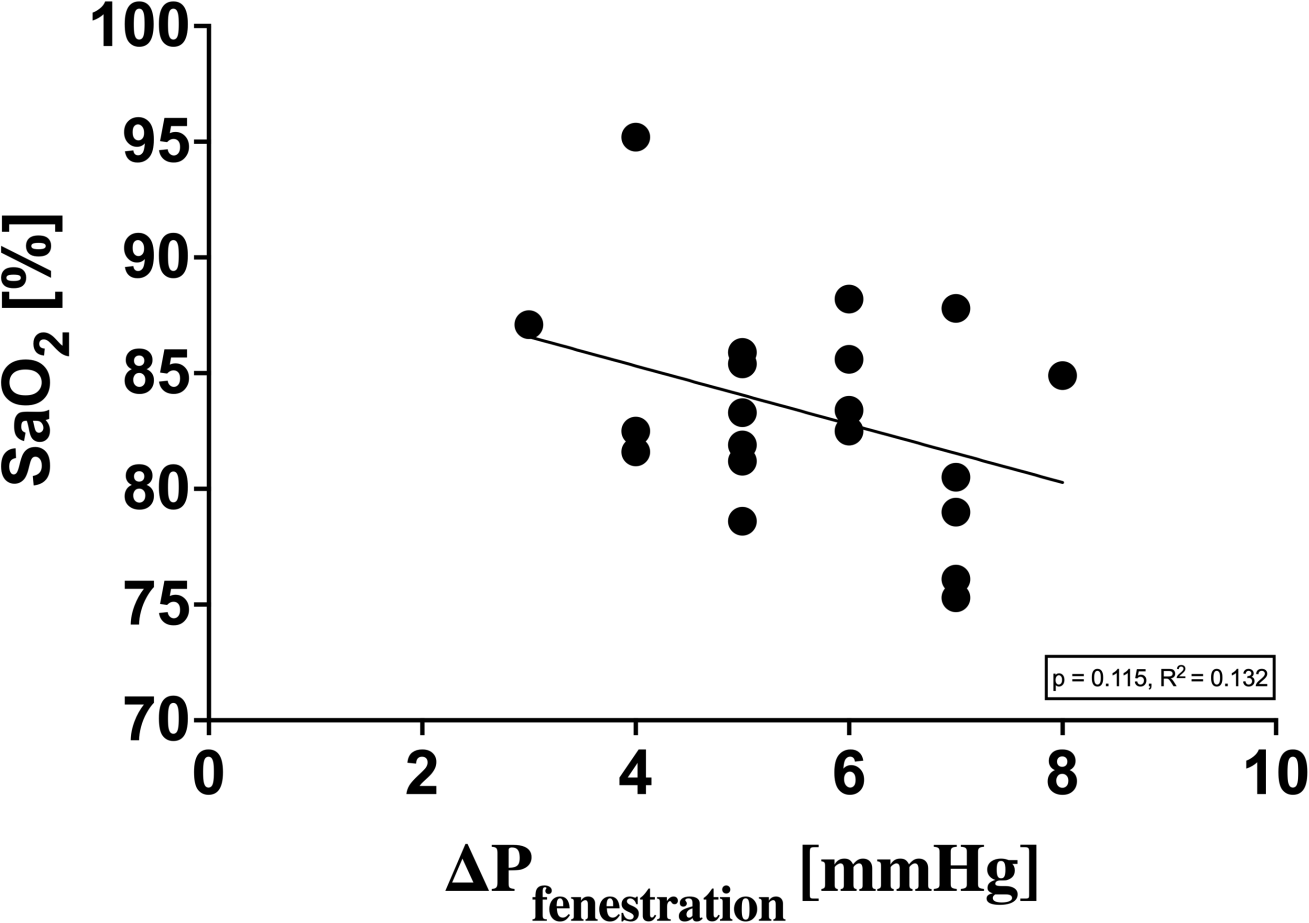
Relationship between SaO_2_ and delta P (Fontan tunnel to atrium).

In seven patients with an open fenestration in the Fontan tunnel, delta P between the venous system and the atrium could be measured. Hypoxia did not lead to a relevant increase in delta P as an indirect indicator of an acute increase in PAP. A clear correlation between peripheral oxygen saturation and delta P could not be shown (Figure 3).

## 4. Discussion

To our knowledge, this is the first study performing a complex fitness-to-fly investigation in a normobaric hypoxia chamber simulating flight conditions in children and adolescents after completing the Fontan procedure with a duration of 3 h.

All patients finished the investigation in good clinical condition and all measured parameters did not show any contraindication for air traveling within a three-hour range for this group. To date, there are no standard values for Fontan patients that can be used for a safe stay on board a passenger aircraft.

Previous studies have already addressed this issue in terms of hypoxic challenge tests (HCT) for different groups of patients. Some were already summarized in reviews by Spoorenberg et al. for children with congenital heart and lung disease in 2016 (19), by Balfour-Lynn et al. for children with lung disease in 2016 (20), and by Herberg et al. for children with pulmonary hypertension in 2020 (18). With a test duration of 5–20 min, these are significantly shorter than the data presented here. Considering the underlying physiology of altitude adaptation, the selected time period seems too short in most cases. Acclimatization to a hypoxic environment has at least two phases. The initial constrictor phase takes several minutes and is followed by a sustained phase. In total, it takes about 30–120 min for the PVR to stabilize. Factors such as neurohumoral mediators, red blood cells, and lung innervation may also influence the response (8). For patients with Fontan physiology, the PVR is one of the central mechanisms for functioning or failing circulation as they lack the subpulmonary ventricle. Accordingly, they have no way to compensate for increasing pulmonary vascular resistance with mechanisms known from healthy subjects (21). If the test duration is too short, the adaptation processes are not completed, and, therefore, a reliable statement on the airworthiness of Fontan patients using HCT cannot be made. The direct measurement of PVR is only possible with invasive methods like a central venous line or in the catheter laboratory so that the interpretation of indirect findings like SaO_2_, SpO_2_, rSO_2_, and lactate as well as echocardiographic values are needed to assess the clinical situation and a potential risk for the patients.

In most HCT investigations, patients are breathing oxygen-reduced air (15.0–15.2%) via a face-mask (13, 22) or sitting in a sealed body plethysmograph (7, 11, 12). This setting indeed seems not feasible for a test duration of more than 30 min.

In 2016, Spoorenberg et al. published fitness-to-fly data for patients with congenital heart disease including changing body positions (seated, lying supine, and standing) combined with different activity levels (walking at 3 and 5 km/h). The test duration here was 40 min. He involved two Fontan patients, again breathing oxygen-depleted air via a face mask (13). Since regular fluid intake is critical for good circulatory function, especially for Fontan patients, a test duration of >30 min would not be practical with such an experimental setup. At the current time, there is no study that shows a comparable structure and complexity over a three-hour investigation period.

The basis for this type of investigation is recommendations, i.e., from the BTS for a fitness-to-fly assessment to provide practical advice for specialists dealing with patients with lung diseases and the still existing uncertainty of families entrusted to their care (4). For patients with congenital heart defects, we do not have any existing guidelines.

Takken et al., Staempfli et al., Garcia et al., and Müller et al. have investigated the effects of altitude on the Fontan circulation but all in connection with physical exertion and thus set a different focus. Nevertheless, these studies may help to better frame the baseline data and are therefore included here (23–26).

### 4.1. Basic monitoring

Since heart rate is influenced by many different factors, a slight increase together with stable blood pressure in all of our patients during the whole investigation time can be interpreted as a stable condition of the patients. There were no signs of physical exhaustion and a relevant need for increased cardiac output. The broad distribution for heart rate is due to the wide age spectrum (3.2–14.7y) and did not correlate with SaO_2_ changes.

### 4.2. Oxygen saturation and blood gas analysis

The average baseline saturation in the present young Fontan cohort was 93 ± 4%. This is in line with data from Takken et al. (94.6% ± 2.9), Staempfli et al. (90% ± 4) at 504 masl, Müller et al. (92% ± 4) (23, 24, 26), and Morimoto et al. (93; 88%–96%), with a similar range of baseline saturation (15). Compared to previous fitness-to-fly data from patients with different underlying diseases, the baseline saturation of Fontan patients is significantly lower [Boosley: SpO_2_ 100% in 71 term and ex-preterm babies (12); Vetter-Laracy: SpO_2_ 99% in 119 babies, term and preterm with and without BPD; Oades: 97.1% in 22 children with cystic fibrosis (27)]. The lower level of oxygen saturation in Fontan patients can be explained by fenestration of the tunnel, severe veno-venous collaterals, and the draining of the coronary sinus in the common atrium, all leading to a desaturation under normoxia and hypoxia (28).

Looking at the course of peripheral oxygen saturation, the largest drop occurs within the first 30 min after hypoxia onset (86% ± 4%). As the process continues, the saturation then stabilizes at a somewhat higher level (Figure 1) but still below baseline saturation. Morimoto reports SpO_2_ values of 88% (78%–95%) at the top of climbing, approximately 20 min after take-off, and a minimum of 86% (78%–95%) after one hour under real flight conditions (hypobaric hypoxia). With HCT investigation times of a maximum of 20 min, these processes cannot be captured and might lead to incorrect conclusions like erroneous bans for patients.

As the drop of SpO_2_ did not show a strong correlation with baseline values, predictability of flight suitability exclusively based on the SpO_2_ at ground level is considerably difficult.

Looking at the capillary saturation measured at 0, 90, and 180 min in hypoxia, there is a difference of up to ±4.7 points compared to SpO_2_ values, probably due to a poor signal caused by restricted peripheral circulation and, consequently, cold hands in some patients and a technically induced deviation between SaO2 and SpO2 from approximately 2%–3% (29). Since this is a known problem, capillary SaO_2_ is certainly the more reliable method. With a correlation of *R*^2 ^= 0.672 between both methods, SpO_2_ still is a good parameter as long as the signal is adequate.

The SaO_2_ values cannot confirm the slight upward trend after initial habituation detected by the continuous oximetry. It remains stable in the follow-up after 180 min without a further decrease. Regardless of all this, stable tissue saturation in the forehead could be documented by NIRS in all patients at any time without significant changes. As expected, there was no correlation to SaO_2_, indicating safe conditions and stable hemodynamics with the maintenance of perfusion of central organ systems even in times of lower SpO_2_ (*R*^2 ^= 0.035).

As a rule, it takes 20 min for a passenger aircraft to reach the maximum cruising altitude. During the ascent there is a slow decrease in pO_2_, so that adaptation to the hypoxic environment can take place slowly. The presented study and the above-cited HCT studies except those by Morimoto et al. expose the participants to 15.0%–15.2% oxygen immediately without any adaptation due to the study setup with normobaric simulated altitude. This might make a difference in oxygen saturation, especially within the first hour. Whether this leads to deviating kinetics of the oxygen saturation must be clarified in a follow-up study.

#### 4.2.1. Lactate and acid-base balance

Rising lactate is an expression of anaerobic metabolic processes, which occurs due to desaturation in the tissue. The assessment of capillary lactate values in our cohort shows an initial increase at T3. This seems to be an expression of acute hypoxia-induced stress. Probably as a sign of stabilization of hemodynamics in the course, lactate does not increase further until T6, comparable to SaO_2_ levels (Table 2B). At T7, no drop in lactate could be observed (*n* = 8). This is most likely due to the short time interval to the end of hypoxia and delayed leaching due to the higher CVP and accumulation of lactate in the tissues over the three-hour period even with only mild desaturation. Stable values for pH, bicarbonate, base excess, and CO on the other hand indicate balanced circulatory conditions at all times. The partially statistically significant differences in Table 2B are not relevant to the clinical condition of the patients.

Stable hemoglobin values in the majority of the patients indicate sufficient drinking quantity during the examination. As described by Zubac et al., the microclimate of airline cabins is dry with a relative humidity of 10%–20% at cruising altitude, and especially in patients with Fontan circulation, this must be addressed before boarding an airplane, as dehydration can lead to a deterioration in circulation and oxygen saturation causing avoidable complications on board (30). This aspect is not considered in the HCT due to the short examination time. However, liquid intake would not be technically possible when breathing through a facemask.

### 4.3. Echocardiography and pulmonary vascular resistance

All patients underwent three echocardiograms during the course and neither showed relevant impairment of systemic ventricle function nor had indirect signs of increasing PVR like rising lactate (Table 3). Whether this can also be applied to older patients with Fontan circulation needs to be investigated in further studies. Qunate et al. recently published the first echocardiography data for older Fontan patients (20.5 years; ±5.4 years) under the same normobaric hypoxia with 15.2% oxygen, comparing normoxia vs. hypoxia at rest and after peak and continuous exercise (31). To our knowledge, no echocardiography parameters have been published from Fontan patients at that young age under hypoxic conditions over a period of 3 h.

#### 4.3.1. Cardiac output

Cardiac function was estimated via VTI and did not show a significant decrease (Table 3). To describe cardiac output, VTI can be multiplied by heart rate as done by Quante et al. They described a decrease in CO by −12% at rest (30 min of adaption sitting on a chair in the hypoxic chamber under the same condition), compared to measurements in normoxia 2 weeks before. In our present younger study group, CO was stable without significant changes over the 3 h study period. Compared to Quante (30 min), the first echocardiography in this study was performed after 90 min in the hypoxic environment. Whether a drop in HZV also occurred within the first 30 min in our cohort and then a recovery until the first echocardiography was not investigated here. Anyhow, this can also be used as an argument for a longer examination duration, especially for Fontan patients. Besides this, it must also be seen in the context of acute exposure due to the study design with full altitude exposure beginning immediately. Comparable with the development of SpO_2_ values, the actual conditions in the aircraft with a slow climb to the final cruising altitude are probably better compensated. However, in conjunction with the lactate values, there is no evidence of an acute hemodynamic problem. In addition, stable CO and lactate values indicate adequate fluid intake during the study period. The broad distribution in standard deviation can be explained by the varying age of the patients and the different anatomical conditions of the ventricles causing a varying CO.

#### 4.3.2. Diastolic function

Quante et al. described a missing augmentation of atrial contraction with a drop of e-wave and a lack of decreasing a-wave. This could be confirmed in our younger cohort and must be interpreted as an expression of hypoxia-related mild diastolic dysfunction. If a long-term stay at a high altitude would lead to an aggravation of ventricular dysfunction or an adaptation with normalization of the values must be clarified. In healthy individuals, it is known that on acute exposure to hypoxia, ventricular diastolic dysfunction is usually prevented by an augmentation of atrial contraction (32).

#### 4.3.3. Pulmonary artery pressure

As already addressed, the PAP plays a key role that determines the function or failure of the Fontan circuit. An estimation of the PAP in healthy individuals with echocardiography is possible by measuring the systolic right ventricular to the right atrial pressure gradient. This was shown by Allemann et al., who investigated 118 healthy children and adolescents 40 h after rapid ascend to 3,450 m. They found a more than two-fold higher PAP compared to a low altitude and an inverse relation to age, resulting in a two-fold larger increase in 6–9 than in 14 to 16-year-old participants (33).

The noninvasive echocardiography estimation of PAP in Fontan patients only works if there is a fenestration between the tunnel and the atrium. In that case, the transpulmonary gradient can be measured via the delta P. As shown by Bouhout et al. in a meta-analysis, a fenestration can effectively reduce pulmonary pressure in the early postoperative period in Fontan patients (34). Whether this could also be helpful in the further course, e.g., for an acute, hypoxia-related change in PVR in connection with high altitude or air travel is conceivable but needs further investigation.

A significant increase in delta P could be excluded by echocardiography in the seven patients with present fenestration in our study. Further, there was no correlation between SaO_2_ and delta P (Figure 3). To what extent this is an indication of worsening diastolic function with an increase in atrial pressure or a lack of rising pulmonary resistance causing this remains an interesting question that cannot be answered with certainty without an invasive measurement of PAP. It is conceivable that veno-venous collaterals also play a role in the sense of a pressure relief of the venous system, especially in the case of above-average desaturation under hypoxia.

The echocardiographic findings are very valuable, underlining the good clinical conditions and subjective well-being of the patients.

## 5. Conclusion

All 21 children finished the investigation without any adverse events.

A fitness-to-fly investigation for patients with Fontan physiology can help to categorize the risk profile and might prevent emergency situations during air travel. The baseline oxygen saturation is not a good marker for the maximum extent of saturation decay in the hypoxic environment of a passenger aircraft.

Regardless of the low oxygen saturation, the patients did not complain of any subjective problems. Objective criteria such as lactate, pH, and base excess as well as tissue saturation in the frontal brain did not show any critical values. The altitude equivalent of 2,500 masl does not lead to relevant impairment in ventricular function. An adequate increase in cardiac output is possible under these conditions. The PVR has a key role in the Fontan circulation, and with regard to physiological adaptation processes, a Hypoxic Challenge Test with a duration of less than 30 min hypoxic exposure might not be sufficient for this special patient group.

From the results of the 21 children examined, it is currently not possible to make any reliable general statement. The flight fitness of Fontan patients, therefore, remains an individual decision of the treating cardiac center together with the patients and their families on the basis of their current state of health. Establishing a sufficient fitness-to-fly protocol for patients with Fontan circulation could help to provide safety for patients, caregivers, and healthcare professionals as well as Airline companies.

## 6. Limitations

Since the investigations took place in a normobaric hypoxia chamber, all results are based on simulated altitude, which is assumed to be equal to real altitude conditions for this special issue. The small number of patients does not allow a fundamental statement for all patients with Fontan circulation. Due to logistic reasons, the children were exposed to a sudden onset of hypoxia, induced by entering the chamber, with 15.2% oxygen content. This does not correspond to the duration of the climb of a passenger aircraft, which lasts on average up to 20 min and thus enables a slow adaptation to the falling effective oxygen content.

## Data Availability

The raw data supporting the conclusions of this article will be made available by the authors, without undue reservation.

## References

[1] Zentner D Celermajer DS Gentles T d’Udekem Y Ayer J Blue GM Management of people with a Fontan circulation: a cardiac society of Australia and New Zealand position statement. Hear Lung Circ. (2020) 29:5–39. 10.1016/j.hlc.2019.09.010 31735685

[2] Plappert L Edwards S Senatore A De Martini A. The epidemiology of persons living with Fontan in 2020 and projections for 2030: development of an epidemiology model providing multinational estimates. Adv Ther. (2022) 39:1004–15. 10.1007/s12325-021-02002-3 34936056 PMC8866255

[3] Rychik J Atz AM Celermajer DS Deal BJ Gatzoulis MA Gewillig MH Evaluation and management of the child and adult with Fontan circulation: a scientific statement from the American heart association. Circulation. (2019) 140:e234–84. 10.1161/CIR.0000000000000696 31256636

[4] Coker RK Armstrong A Church AC Holmes S Naylor J Pike K BTS clinical statement on air travel for passengers with respiratory disease. Thorax. (2022) 77:329–50. 10.1136/thoraxjnl-2021-218110 35228307 PMC8938676

[5] Withers A Wilson AC Hall GL. Air travel and the risks of hypoxia in children. Paediatr Respir Rev. (2011) 12:271–6. 10.1016/j.prrv.2011.02.002 22018043

[6] Lee AP Yamamoto LG Relles NL. Commercial airline travel decreases oxygen saturation in children. Pediatr Emerg Care. (2002) 18:78–80. 10.1097/00006565-200204000-00004 11973496

[7] Vetter-Laracy S Osona B Peña-Zarza JA Gil JA Figuerola J. Hypoxia challenge testing in neonates for fitness to fly. Pediatrics. (2016) 137:e20152915. 10.1542/peds.2015-2915 26908703

[8] Wilkins MR Ghofrani HA Weissmann N Aldashev A Zhao L. Pathophysiology and treatment of high-altitude pulmonary vascular disease. Circulation. (2015) 131:582–90. 10.1161/CIRCULATIONAHA.114.006977 25666980

[9] Penaloza D Arias-Stella J. The heart and pulmonary circulation at high altitudes: healthy highlanders and chronic mountain sickness. Circulation. (2007) 115:1132–46. 10.1161/CIRCULATIONAHA.106.624544 17339571

[10] Becker K Uebing A Hansen JH. Pulmonary vascular disease in Fontan circulation-is there a rationale for pulmonary vasodilator therapies? Cardiovasc Diagn Ther. (2021) 11:1111–21. 10.21037/cdt-20-431 34527537 PMC8410499

[11] Naqvi N Doughty VL Starling L Franklin RC Ward S Daubeney PEF Hypoxic challenge testing (fitness to fly) in children with complex congenital heart disease. Heart. (2018) 104:1333–8. 10.1136/heartjnl-2017-312753 29444807

[12] Bossley CJ Cramer D Mason B Hayward A Smyth J McKee A Fitness to fly testing in term and ex-preterm babies without bronchopulmonary dysplasia. Arch Dis Child Fetal Neonatal Ed. (2012) 97:199–203. 10.1136/adc.2011.212001 21785127

[13] Spoorenberg ME Hulzebos EHJ Takken T. Feasibility of hypoxic challenge testing in children and adolescents with congenital heart and lung disease. Aerosp Med Hum Perform. (2016) 87:1004–9. 10.3357/AMHP.4580.2016 28323585

[14] Spoorenberg ME Hulzebos EHJ Takken T. Feasibility of hypoxic challenge testing in children and adolescents with congenital heart and lung disease. Aerosp Med Hum Perform. (2017) 87:1004–9. 10.3357/amhp.4580.2016 28323585

[15] Morimoto Y Ohuchi H Kurosaki K Nakai M. Exercise-induced hypoxia predicts hypobaric hypoxia during flight in patients after Fontan operation. Int J Cardiol. (2021) 325:51–5. 10.1016/j.ijcard.2020.09.069 33010380

[16] Harinck E Hutter PA Hoorntje TM Simons M Benatar AA Fischer JC Air travel and adults with cyanotic congenital heart disease. Circulation. (1996) 93:272–6. 10.1161/01.CIR.93.2.272 8548899

[17] Smith D Toff W Joy M Dowdall N Johnston R Clark L Fitness to fly for passengers with cardiovascular disease. Heart. (2010) 96(Suppl 2):10. 10.1136/hrt.2010.203091 20644218

[18] Herberg U Knies R Müller N Breuer J. Altitude exposure in pediatric pulmonary hypertension-are we ready for (flight) recommendations? Cardiovasc Diagn Ther. (2021) 11:1122–36. 10.21037/cdt-20-494 34527538 PMC8410490

[19] Spoorenberg ME van den Oord MHAH Meeuwsen T Takken T. Fitness to fly testing in patients with congenital heart and lung disease. Aerosp Med Hum Perform. (2015) 87:54–60. 10.3357/amhp.4408.2016 26735234

[20] Balfour-Lynn IM. Hypoxic challenge test for airflight in children with respiratory disease. Paediatr Respir Rev. (2017) 21:62–4. 10.1016/j.prrv.2016.05.002 27427310

[21] Netzer NC Strohl KP Högel J Gatterer H Schilz R. Right ventricle dimensions and function in response to acute hypoxia in healthy human subjects. Acta Physiol. (2017) 219:478–85. 10.1111/apha.12740 27332955

[22] Kobbernagel HE Nielsen KG Hanel B. Hypoxic challenge test applied to healthy children: influence of body positions and exertion on pulse oximetric saturation. Arch Dis Child. (2013) 98:602–6. 10.1136/archdischild-2012-302763 23814087

[23] Takken T Evertse A de Waard F Spoorenburg M Kuijpers M Schroer C Exercise responses in children and adults with a Fontan circulation at simulated altitude. Congenit Heart Dis. (2019) 14:1005–12. 10.1111/chd.12850 31602790 PMC7003737

[24] Staempfli R Schmid JP Schenker S Eser P Trachsel LD Deluigi C Cardiopulmonary adaptation to short-term high altitude exposure in adult Fontan patients. Heart. (2016) 102:1296–301. 10.1136/heartjnl-2016-309682 27217067

[25] Garcia JA McMinn SB Zuckerman JH Fixler DE Levine BD. The role of the right ventricle during hypobaric hypoxic exercise: insights from patients after the Fontan operation. Med Sci Sports Exerc. (1999) 31:269–76. 10.1097/00005768-199902000-00011 10063817

[26] Müller N Herberg U Jung T Breuer J Härtel JA. Adequate Exercise Response at Artificial Altitude in Fontan Patients. Front Pediatr. (2022) 10:1–13. 10.3389/fped.2022.947433 PMC9433899 36061398

[27] Oades P Buchdahl R Bush A. Predictions of hypoxaemia at high altitude in children with cystic fibrosis. Br Med J. (1994) 308:15–8. 10.1136/bmj.317.7161.780 8298345 PMC2539154

[28] Gewillig M. The fontan circulation. Heart. (2005) 91:839–46. 10.1136/hrt.2004.051789 15894794 PMC1768934

[29] Jubran A. Pulse oximetry. Crit Care. (2015) 19:1–7. 10.1186/s13054-015-0984-8 26179876 PMC4504215

[30] Zubac D Stella AB Morrison SA. Up in the air: evidence of dehydration risk and long-haul flight on athletic performance. Nutrients. (2020) 12:1–15. 10.3390/nu12092574 PMC7551461 32854320

[31] Quante H Müller N Härtel JA Jung T Manunzio U Breuer J Systemic ventricular function in Fontan patients at rest and after exercise at altitude. Front Pediatr. (2023) 10:1–11. 10.3389/fped.2022.1084468 PMC9853047 36683788

[32] Allemann Y Rotter M Hutter D Lipp E Sartori C Scherrer U Impact of acute hypoxic pulmonary hypertension on LV diastolic function in healthy mountaineers at high altitude. Am J Physiol Hear Circ Physiol. (2004) 286:856–62. 10.1152/ajpheart.00518.2003 14604853

[33] Allemann Y Stuber T de Marchi SF Rexhaj E Sartori C Scherrer U Pulmonary artery pressure and cardiac function in children and adolescents after rapid ascent to 3,450 m. Am J Physiol Hear Circ Physiol. (2012) 302:2646–53. 10.1152/ajpheart.00053.2012 22523248

[34] Bouhout I Ben-Ali W Khalaf D Raboisson MJ Poirier N. Effect of fenestration on Fontan procedure outcomes: a meta-analysis and review. Ann Thorac Surg. (2020) 109:1467–74. 10.1016/j.athoracsur.2019.12.020 31987825

